# Osteopontin-Enhanced Hepatic Metastasis of Colorectal Cancer Cells

**DOI:** 10.1371/journal.pone.0047901

**Published:** 2012-10-24

**Authors:** Jianjin Huang, Chi Pan, Hanguang Hu, Shu Zheng, Ling Ding

**Affiliations:** 1 Department of Medical Oncology, Second Affiliated Hospital, Medical School of Zhejiang University, Hangzhou, China; 2 Cancer Institute, Second Affiliated Hospital, Medical School of Zhejiang University, Hangzhou, China; University of Kentucky College of Medicine, United States of America

## Abstract

Liver metastasis is a major cause of mortality from colorectal cancer (CRC). However, mechanisms underlying this process are largely unknown. Osteopontin (OPN) is a secreted phosphorylated glycoprotein that is involved in tumor migration and metastasis. The role of OPN in cancer is currently unclear. In this study, OPN mRNA was examined in tissues from CRC, adjacent normal mucosa, and liver metastatic lesions using quantitative real-time PCR analysis. The protein expression of OPN and its receptors (integrin αv and CD44 v6) was detected by using an immunohistochemical (*IHC*) method. The role of OPN in liver metastasis was studied in established colon cancer Colo-205 and SW-480 cell lines transfected with sense- or antisense-OPN eukaryotic expression plasmids by flow cytometry and cell adhesion assay. Florescence redistribution after photobleaching (FRAP) was used to study gap functional intercellular communication (GJIC) among OPN-transfected cells. It was found that OPN was highly expressed in metastatic hepatic lesions from CRC compared to primary CRC tissue and adjacent normal mucosa. The expression of OPN mRNA in tumor tissues was significantly related with the CRC stages. OPN expression was also detected in normal hepatocytes surrounding CRC metastatic lesions. Two known receptors of OPN, integrin αv and CD44v6 proteins, were strongly expressed in hepatocytes from normal liver. CRC cells with forced OPN expression exhibited increased heterotypic adhesion with endothelial cells and weakened intercellular communication. OPN plays a significant role in CRC metastasis to liver through interaction with its receptors in hepatocytes, decreased homotypic adhesion, and enhanced heterotypic adhesion.

## Introduction

Colorectal cancer (CRC) continues to be the second leading cause of cancer-related deaths in the United States [1]. Approximately 50% of CRC patients have metastases in the liver, but only 10%–20% of these patients can undergo surgical resection. The 5-year survival rate following surgical resection of metastatic tumor is only 30%–40% [2], [3].The molecular mechanisms underlying CRC metastasis are not completely understood but recent evidences suggest that osteopontin (OPN) plays a role in regulating colon cancer cell metastasis [4].

OPN is a phosphoglycoprotein which is originally isolated from mineralized bone matrix [5], also is frequently secreted by different types of cancer cells, essential for growth and metastasis of breast [6], prostate [7], [8], hepatic [9], melanoma [10], and other tumors [11]. OPN signals through cell adhesion molecules, such as integrins and CD44 [12], [13]. Gene-profiling studies have identified a correlation between advanced and metastatic colorectal tumors and high expression of OPN [14]. A major influence of OPN on metastatic potential is through cellular attachment and migration across extracellular matrix proteins22. However, the functional roles and underlying mechanisms of OPN in colorectal metastasis have not been established.

Here, we investigated the role of OPN in colon metastasis to liver and the mechanisms by which OPN promotes colon cancer metastatic activity.

## Materials and Methods

### Patients

Tumor specimens from 44 cases of CRC and 20 patients with liver metastasis at the Second Affiliated Hospital of Zhejiang University were obtained fresh from surgical resections with prior consent. The median age of the patients was 57 years at operation, ranging from 25 to 84. Pathological characteristics including tumor location, stage, and differentiation was recorded. This study has obtained human research ethics approval from the Ethics Committee of the Second Affiliated Hospital, School of Medicine, Zhejiang University.

### Cell Culture

Two colorectal adenocarcinoma cell lines Colo-205 and SW480 cells were obtained from the Cancer Institute of Zhejiang University and maintained in RMPI-1640 with 10% calf serum at 37°C with 5% CO_2_.

### Quantitative Real-time PCR

Tissue samples were snap-frozen immediately after surgical resection. The total RNA was isolated using Trizol (Invitrogen) according to the manufacture’s instructions. RNA integrity was examined by gel-electrophoresis with 1% formaldehyde gel. 2.0 µg total RNA was reversely transcribed with SUPER SCRIPT II RTPCR kit (Invitrogen). Real-time PCR was performed in a final volume of 50 µl containing 1 µl RT transcript, 5 µM of each primer, 0.5 unit of AmpliTaq DNA polymerase (Invitrogen). As an internal positive control, real-time PCR analysis was performed on the GAPDH gene in parallel. The following primers were used: OPN forward primer 5′-CAA ATACCCAGATGCTGTGGC-3′; OPN reverse primer 5′-TCCTGGCTGTCCACATGGTC-3′; GAPDH forward primer 5′-CTTAGCACCCCTCCCCAAG-3′; GAPDH reverse primer 5′- GATGTTCTGGAGAGCCCCG-3′. OPN TaqMan probe 5′-TGACCCTACTCAGAAGCAGAATCTCCTAGCC-3′; GAPDH TaqMan probe 5′-CATGCCATCACTGCCACCCAGAAGA-3′. PCR amplifications were carried out in a 96-well reaction plate format and the fluorescence signals were detected with a PE Applied Biosystems 7700 Sequence Detector (Perkin-Elmer). The fluorescence signals were translated into C_T_ values (cycle threshold; corresponding to the cycle number at which a significant increase in the fluorescence signal was first detected). The level of mRNA expression of OPN = OPN C_T_ value/GAPDH C_T_ value.

PCR products were electrophoresed on agarose gels and stained with ethidium bromide and photographed. PCR products of the expected size were cloned into a pGEM-T-Easy vector (Promega) and the sequences of the resulting plasmids were confirmed.

### Antisense- and Sense-OPN Eukaryotic Expression Plasmids

To obtainan anti-sense OPN expression plasmid, we generated a 201 bp cDNA fragment (from 210 to 368) using PCR with OPN primers as mentioned above. cDNA fragment was then cloned into the *EcoR* I site of the pcDNA 3.1(+). The reverse direction of the cloned cDNA were confirmed by DNA sequencing (The vector is designated OPN-antisense).

The open reading frame (ORF) of OPN was obtained by RT-PCR. OPN primer F: 5′- CCG ACC AAG GAA AAC TCA CT - 3′, R:5′- CTC CTT TTA ATT GAC CTC AG - 3′. The 974 bp PCR products were cloned into a pGEM-T-Easy vector and sequenced on both strands using the ABI PRISM™ 377 DNA Sequencer (Perkin-Elmer). The ORF of the OPN was then cloned into a eukaryotic expression vector pcDNA 3.1(+) to generate pcDNA 3.1-OPN (ORF). (The vector is designated OPN-sense).

### Stable Expression Cell Lines

The plasmids of OPN-antisense, OPN-sense and pcDNA3.1(+)were linearlized with *Bgl?* digestion for 4 hr. DNA transfection was performed using the Superfect Transfection Reagent (Qiagen) following the manufacturer’s instructions. After 48 hr, transfected cells were selected with G418 for 3 days. Selected clones were pooled and expanded. The established cell lines were confirmed with RT-PCR and Western bolt analysis.

### *In Situ* mRNA Hybridization (*ISH*)

Specific probes complementary to the OPN mRNA were designed based on GenBank J04765 (Human osteopontin mRNA, complete cds). These oligonucleotide sequences have 100% homology with the OPN gene and minimal homology with other mammalian gene sequences as searched in GenBank by blast. DIG RNA Label Kit (SP6/T7) (Roche) was utilized to generate both sense and antisense. Paraffin-embedded tissues were sectioned at 4 μ m. The slides were deparaffinized and treated with 0.2% M. HCl for 20 min. After digested with proteinase K, slides were incubated with pre-hybridization solution (containing 500 µg/ml poly (A)) at 42°C for 30 min in a moisture chamber. The solution was replaced by hybridization solution (containing 0.3 µg/ml sense or anti-sense RNA single strand probe respectively, 50% formamide, 10% dextransulfate, and 2×SSC), and hybridized at 42°C overnight. After washed with 2×SSC and 1×SSC for 15 min succession, the slides were blocked with PBS containing 5% bovine serum albumin for 10 min, incubated with biotin-labeled mouse-anti-digoxin antibody for 30 min and then with alkaline phosphoesterase-affinitin for 30 min. NBT-BCIP was used to stain slides in pH 10 substrate buffer. After dehydration, slides were mounted with aqueous mounting media (Sigma).

### Immunohistochemistry Analysis

Rat anti-human osteopontin monoclonal antibody (Chemicon, Billerica, MA) was used for *IHC*. All sections (5 µm-thick) from formalin-fixed paraffin-embedded tissue blocks were mounted on gelatin coated glass slides. To enhance antigen exposure, the slides were treated with 1×EDTA at 98°C for 10 min for antigen retrieve. The slides were incubated with endogenous peroxidase blocking solution to inhibit endogenous peroxidase, then were incubated with the primary antibody (either anti-OPN mouse monoclonal antibody (Akm2A1; Santa Cruz Biotechnology, Santa Cruz, CA) at a dilution of 1∶200, or integrin αv monoclonal antibody (P2W7; Santa Cruz Biotechnology, Santa Cruz, CA) at a dilution of 1∶200, or CD44v6 monoclonal antibody (MAB-0038; Maixin, Fujian, China) at a dilution of 1∶100) at room temperature for 60 min. After rinsed with Tris-buffered saline, the slides were incubated for 15 min with biotin-conjugated secondary antibody, washed and then incubated with enzyme conjugate HRP (horseradish peroxidase)-streptavidin. Freshly prepared DAB (Zymed, South San Francisco, CA) was used as substrate to detect HRP. Finally, slides were counterstained with hematoxylin and mounted with aqueous mounting media.

### Cell Adhesion Assay

Monolayer Colo-205 cells and SW-480 cells were maintained in Ø 35 mm dishes and normal medium until 70%–80% confluence. Attached cells were then overlaid on the monolayer cells, mixed well and incubated for different periods of time.

Floating cells were collected after incubation for 10 min, 20 min, 30 min and 40 min, and the number was counted using a hemocytometer. Cell adhesion ratio was then calculated: adhesion ratio = (sum of cells added- suspended cells)/sum of sample cells. Homotypic adhesion assay was performed with same cell lines, each of them between navel vessel endothelium cells ecv 304 cells was used respectively in heterotypic adhesion assay.

### Flow Cytometry Analysis

1×10^6^/ml cell samples were suspended thoroughly in PBS, mixed with 20 µl CD44-FITc antibody, controlled with 20 µl IgG1/2a antibody, cell incubated with Ab at RT for 20 min. Rinsed with PBS, mixed with 1% paraformal-dehyde, then detected with FAC Scan (BD company, Cell Quest TM Version 3.3 software).

### Gap-fluorescence Redistribution After Photobleaching (Gap-FRAP) Method

Cells of 80% confluence were examined for fluorescence redistribution after photobleaching. Cells rinsed with Hanks (Ca^2+^, Mg^2+^), incubated 15 min with 10 µg/ml 5(6)-carboxyfluorescein diacetate (CFDA) in 37°C, 5% CO_2_. After rinsed by PBS, a cell adjacent to other cells was selected and its fluorescence was photobleached under LEICA TCS-SP laser co-focus microscope (Argon ion laser beam, 518 nM, Time/lampse program for photobleaching and scan, Physiology software for image and data). Digital images of the fluorescent emission excited by weak laser pulses were recorded at regular intervals for 12 min (scanning period 1 minute before and after photobleaching) and stored for subsequent analysis. In each experiment, one labeled, isolated cell was left unbleached as a reference for the loss of fluorescence due to repeated scanning and dye leakage, and an isolated, bleached cell served as a control.

### Statistical Analysis

Data are expressed as mean±SEM. Paired-samples *t* test, independent-samples *t* test and one-way ANOVA were used. Computations were carried out using SPSS 13.0 software. *P* values less than 0.05 were considered significant.

## Results

### OPN Expression in Primary CRC, CRC Metastatic Lesions in Liver, and Normal Tissues

Based on quantitative real-time PCR, C_T_ values are in inverse proportion to the log value of the original copy numbers of target sequence. We found that OPN mRNA was expressed in 44 cases of CRC tissues but at different levels; the maximum C_T_ value was 1.47 and the minimum value was 0.85, 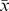
±*s* = 1.15±0.14. When paired with adjacent normal samples, the maximum C_T_ value was 1.75 and the minimum value was 0.94, 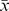
±*s* = 1.32±0.18. (paired-samples *t* test, *t* = 5.81, *P* = 0.000). The OPN mRNA expression was significantly increased in primary CRC tissue compared to neighboring normal tissues.

**Figure 1:**
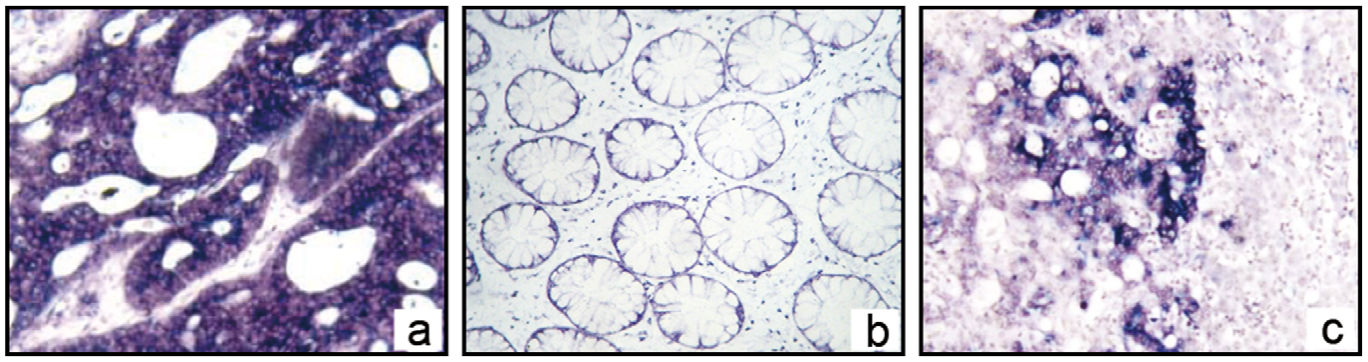
OPN mRNA location in CRC and its liver metastasis with in situ mRNA hybridization. a, In *ISH*, the positive stain presents blue-purple under microscope. The OPN mRNA, which indicates a positive signal, was found in cytoplasm of CRC cells in primary lesions (×400). **b,** The stain in adjacent normal colorectal tissues was negative (×400). **c,** OPN mRNA was found in cytoplasm of CRC cells liver metastatic tissue. Adjacent normal liver tissues stains negative (×100).

In 20 of examined liver metastatic tissues from CRC, the maximum C_T_ value was 1.30 and the minimum value was 0.92, 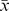
±*s* = 1.07±0.10. Compared to the primary cancer, the OPN mRNA expression in liver metastatic tissues was higher (independent-samples *t* test, *t* = 2.12, *P* = 0.038).

Based on the Dukes stage, seven of total 44 CRC patients were at stage A and the OPN mRNA mean C_T_ value was 1.49±0.38, 1.21±0.28 for stage B(17 patients), 1.11±0.29 for stage C(18 patients ),and 0.92±0.32 for stage D(2 patients). The expression level of OPN mRNA in CRC tissues at early stage was significantly higher than which at advanced stage (one-way ANOVA, *P* = 0.031).

We then used *ISH* analysis to further assess OPN mRNA expression and localization. In this analysis, the positive appears blue-purple under the microscope. OPN mRNA, which appears signal coloration, was found in the cytoplasm of CRC cells in both the primary lesions and liver metastatic tissue. The staining signal was not detected in adjacent normal colorectal and normal liver tissues (*Fig. 1*).

**Figure 2:**
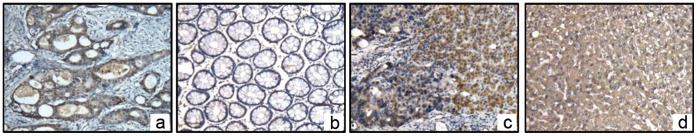
OPN protein localization in the CRC and its liver metastasis visualized by immunohistochemistry. a In *IHC*, OPN proteins (brown positive stain) were localized in cytoplasm of CRC cells in primary lesions(x200). **b** Adjacent normal colorectal tissues were negative(x200). **c** Positive stain was found both in CRC cells and adjacent normal hepatocytes in liver metastatic tissues(x100). **d** As controls, OPN proteins stain in normal liver tissues without CRC metastasis was negative(x100).

Using an immunohistochemical method, we further confirmed that OPN protein was expressed in the cytoplasm of CRC cells in both the primary lesion and metastatic tissues. The brown positive stains were also found in normal hepatocytes around the metastatic tissue. Adjacent normal colorectal tissues exhibited no signal. As control, OPN proteins stain in normal liver tissues without CRC metastasis *(* *Fig. 2* *)*.

### Expression of OPN Receptors-integrin αv and CD44v6 Staining in Normal Hepatocytes

**Figure 3:**
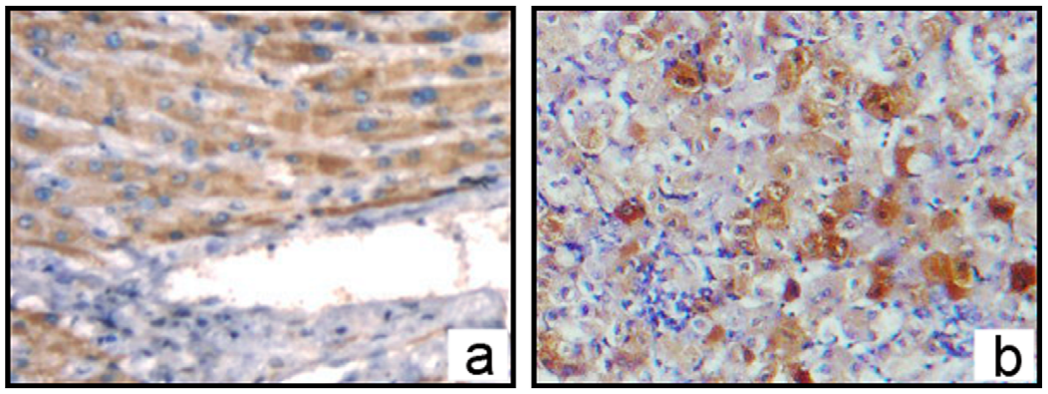
Expression of integrin αv and CD44v6 in normal hepatocytes with Immunohistochemistry. a, In *IHC*, integrin αv proteins (brown positive stain) were localized in cytoplasm of hepatocytes (×400). **b,** CD44v6 proteins (brown positive stain) were localized in cytoplasm of hepatocytes (×400).

OPN has been shown interacting with a number of different integrins via the RGD sequence, including αvβ3, αvβ1 and αvβ5. CD44v6 is a splice variant of CD44 (CD44v) could also bind to OPN and likely promotes cancer cell adherence to the vascular endothelium and the base membranes, then enhances the invasion and metastasis of colonic carcinomas. To verify these receptors in normal liver, we assayed for integrin αvβ1 and CD44v6 by IHC. We found that both integrin αvβ1 and CD44v6 were stained positively in hepatocytes in normal liver tissues *(* *Fig. 3* *).*

### Expression of CD44 on Cell Membrane by Flow Cytometry

CD44 plays a major role in cell-cell adhesion, cell-substrate interaction, lymphocyte homing, and tumor metastasis. Recent studies have shown that high expression of CD44 in certain types of tumors is associated with the hematogenic spread of cancer cells.

We detected CD44 expression in transferred cell lines using flow cytometry. We found that after down-regulation of OPN expression, CD44 expression on Colo-205-OPN-antisense cell membranes was decreased from 54.28±0.11 to 35.35±0.24 in Colo-205, *P*<0.01. In contrast, the value was increased in SW-480- OPN-sense cells in which OPN expression were up-regulated from 2.65±0.21 to 33.12±0.15, *P*<0.01.

### Homotypic and Heterotypic Cell Adhesion

Cell adhesion is an important factor during cancer metastasis. The detachment of cancer cells from their parent tumors is an initial event in metastasis and is related to homotypic adhesion decreasing. Once tumor cells escape from the primary tumor, they interacts with the preexisting host basement membranes at a diverse stages during the metastatic cascade. Heterotypic adhesion helps cancer cells complete this whole process.

We detected the homotypic and heterotypic adhesion ability in Colo-205-OPN-antisense and SW-480-OPN-sense separately. Navel vessel endothelium cells ecv 304 was used as target cells in heterotypic adhesion. Homotypic adhesion ability was enhanced after OPN-antisense transfection in Colo-205, but was weakened in SW-480-OPN-sense. In contrast, heterotypic adhesion ability was weakened in Colo-205-OPN-antisense, but was enhanced in SW-480-OPN-sense. *(data not shown)*.

### Gap Junctional Intercellular Communication (GJIC) with Gap-FRAP

GJIC consists of intercellular exchange of low molecular weight molecules, and is the only means for direct contact between cytoplasms of adjacent animal cells. Disturbances of GJIC have been associated with many pathological conditions, such as carcinogenesis or hereditary illness [15], also may facilitate the release of a potentially neoplastic cell from the controlling of the surrounding tissue, leading to tumour promotion [16].

**Figure 4:**
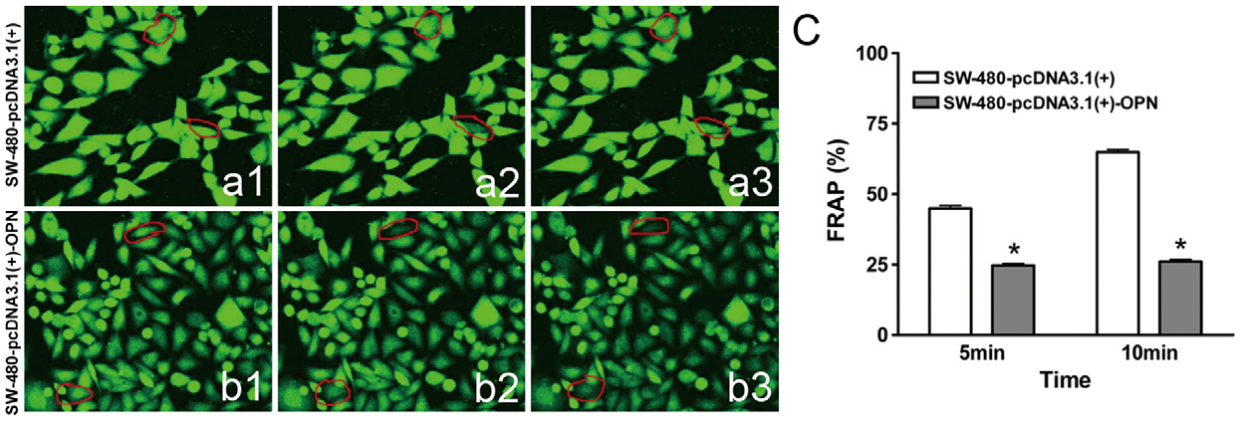
Gap junctional intercellular communication (GJIC) with gap-FRAP in SW-480-pcDNA3.1(+) and SW-480-pcDNA3.1(+) OPN cells. a1, uniform fluorescence intensity before photobleaching in SW-480-pcDNA3.1(+). **a2,** weakened fluorescence intensity after photobleaching in SW-480-pcDNA3.1(+). **a3,** fluorescence redistribution after 10minutes in SW-480-pcDNA3.1(+). **b1,** uniform fluorescence intensity before photobleaching SW-480-pcDNA3.1(+) OPN cells. **b2,** weakened fluorescence intensity after photobleaching SW-480-pcDNA3.1(+) OPN cells. **b3,** faint fluorescence redistribution after 10minutes SW-480-pcDNA3.1(+) OPN cells. **c,** The percentage of fluorescence redistribution after photobleaching (FRAP%) in SW-480-pcDNA3.1(+) and SW-480-pcDNA3.1(+) OPN cells**.** After 5 minutes of photobleaching, the percentage of fluorescence redistribution after photobleaching (FRAP%) 24.65±4.08% in SW-480-pcDNA3.1(+) -OPN compared 44.74±6.23% in SW-480-pcDNA3.1(+)(*t* = 17.07, *P*<0.001), and after 10 minutes, the FRAP% was still be inhibited at 25.98±4.48% in SW-480-pcDNA3.1(+) -OPN while it was redistributed to 64.92%±5.39% in the SW-480-pcDNA3.1(+) cells (*t* = 35.16, *P*<0.001).

We used gap-FRAP to measure GJIC in OPN transfected cells (SW-480-pcDNA3.1(+)-OPN) while Colo-205 were semi-suspension cells which were not appropriated for this method. Cell signal exchange was impaired and GJIC function was inhibited. Compared with control cells (vector transfected SW-480-OPN cells, SW-480-pcDNA3.1(+)), fluorescence was not redistributed well in SW-480-OPN-sense cells after both 5 minutes and 10 minutes of photobleaching. After 5 minutes of photobleaching, the percentage of fluorescence redistribution after photobleaching (FRAP%) was 24.65±4.08% in SW-480-pcDNA3.1(+)-OPN compared 44.74±6.23% in SW-480-pcDNA3.1(+)(*t* = 17.07, *P*<0.001), and after 10 minutes, the FRAP% was still be inhibited at 25.98±4.48% in SW-480-pcDNA3.1(+)-OPN while it was redistributed to 64.92%±5.39% in the SW-480-pcDNA3.1(+) cells (*t* = 35.16, *P*<0.001) (*Fig. 4* ). These results indicate that OPN may inhibit GJIC function and impaired signal exchange among these transfected SW-480 cells.

## Discussion

Human colon cancer affects nearly 150,000 patients and 60,000 deaths in the United States per year. The liver is the primary extra-colonic site for CRC metastasis and represents the most common location and clinical presentation for recurrent disease in patients who fail locoregioal therapy [17]. There are few tumor markers that have clinical utility in the management of CRC metastasis. Even the application of the most widely used marker, carcinoembryoni antigen (CEA), has recently been called into question [18]. OPN is a candidate gene of metastasis in CRC. With the application of gene expression profiling technology and pooled sample expression profiling [19], [20], OPN has been identified as the leading candidate clinical marker of CRC progression. We therefore investigated the role of OPN in regulating hepatic end-organ metastasis.

Using quantitative real-time, we verified that OPN was highly expressed in metastatic hepatic lesions from CRC compared to primary CRC tissue and adjacent normal mucosa. In addition, the expression of OPN mRNA in tumor tissues was significantly related with the CRC stages. These results implicate that OPN is a potential marker for tumor invasion and hepatic metastasis of CRC.

Notably, OPN mRNA was found in the cytoplasm of CRC cells by *in situ* hybridization, but not in their adjacent normal tissues or in the surrounding normal tissues of livers. Immunohistochemical assay showed that the OPN protein also exists obviously in the cytoplasm of adjacent normal hepatocytes surrounding CRC tissues, and that the expression of OPN protein in normal hepatic tissue is negative. However, we detected two receptors of OPN, integrinαv and CD44v6, in normal hepatic tissue. Therefore, we hypothesize that when CRC tumor cells depart from primary lesions, the portal vein which is a necessary circumfluence vein, allows the cancer cells to pass through the liver. The tumor cells containing OPN could accumulate in the liver through an interaction between a ligand and a receptor.

We examined two different cell lines to investigate the role of OPN in regulating metastasis. The Colo-205 cell line was transfected with an OPN-antisense eukaryotic plasmid to inhibit OPN protein expression in this cell line. SW-480 cells were transfected with an OPN-sense eukaryotic plasmid, so that the OPN protein is expressed in this cell line.

After cell transfection, Colo-205-OPN-antisense transfected cells clustered (data not shown), homotypic adhesion was enhanced in these cells in comparison to the Colo-205-pcDNA3.1(+). However the adhesion ability was weakened in SW-480-OPN-sense cells, and the mobility was enhanced in these cells expressing OPN (data not shown). At the same time, we investigated the heterotypic adhesion with ecv304 as target cells, heterotypic adhesion ability was weakened in Colo-205-OPN-antisense cells and was enhanced in SW480-OPN-sense cells. These results suggest that OPN decreases the homotypic adhesion among CRC cells and enhances the heterotypic adhesion ability between CRC cells and endothelium cells.

Invasion and metastasis are important characteristics of malignant tumors. The whole process of metastasis includes angiogenesis adhesion, degradation, movement, and reattachment [21]. Adhesion factors are essentially involved in the process. OPN is probably one of numerous prognostic factors related metastasis of CRC.

Because of OPN, homotypic adhesion ability is weakened in CRC cells and the cells to depart from primary site more easily, and enter the blood circulation. The first step of metastasis is onset. However, the CRC cells are more easily to invade ECM (the extra cellular matrix) when heterotypic adhesion is enhanced. These two steps are important to metastasis in malignant cancer.

GJIC has been speculated to be a necessary, if not sufficient, biological function of metazoan cells in regulation of growth control, differentiation and apoptosis of normal progenitor cells [22]. Intercellular communication in many organs is maintained via intercellular gap junction channels composed of connexins, a large protein family with a number of isoforms. This GJIC allows for the propagation of action potentials (e.g. in brain, heart), and the transfer of small molecules which may regulate cell growth, differentiation and function. The latter has shown to be involved in cancer growth: reduced GJIC often is associated with increased tumor growth or with the process of de-differentiation. Fluorescence redistribution after photobleaching (FRAP) was adopted to observe the recovery of fluorescence intensity in the bleached cells and therefore to estimate intercellular communication by gap junctions.

In our study, fluorescence redistribution after photobleaching was weakened in SW-480- OPN-sense cells. GJIC function was inhibited and signal exchange were impaired, So these tumor cells are more easily detached from primary lesions to initiate the first step of metastasis incidently.

Based on studies of the correlated mechanisms between expression of OPN and metastasis in combination with the pertinent literatures, we concluded that a potential mechanism of OPN promoting CRC liver metastasis is as follows: CRC cells express OPN, the homogeneity adherence ability are decreased, the function of GJIC is inhibited, the capability of invasion and movement is increased, which makes it easy for the CRC cells to depart from primary lesion, get into circulation and initiate the procedure of metastasis. At the same time, due to the OPN, the expression of CD44, a metastasis-related factor, is also increased. The heterotypic adhesion between CRC cells, ECM and vascular endothelial cell is enhanced. In the course of portal vein refluence, OPN allows cancer cells to invade peripheral vessels, settling down and planting in the liver. Furthermore, the receptors of OPN, CD44v6 and integrin αv were found in liver. The interaction between ligands and receptors could explain the liver-cling of CRC cells.

These results suggest that OPN is one of the important factors in tumor infiltration and metastasis. The effect on homotypic and heterotypic adhesion in CRC cells of OPN is essential in the process of liver metastasis.

## References

[1] Jemal A, Bray F (2011) Center MM, Ferlay J, Ward E, et al (2011) Global cancer statistics. CA Cancer J Clin 61: 69–90. 21296855 10.3322/caac.20107

[2] Ruers T, Bleichrodt RP (2002) Treatment of liver metastases, an update on the possibilities and results. Eur J Cancer 38: 1023–1033. 11978527 10.1016/s0959-8049(02)00059-x

[3] Siegel RL, Jemal A, Thun MJ, Hao Y, Ward EM (2008) Trends in the incidence of colorectal cancer in relation to county-level poverty among blacks and whites. J Natl Med Assoc 100: 1441–1444. 19110912 10.1016/s0027-9684(15)31544-3

[4] Yeatman TJ, Chambers AF (2003) Osteopontin and colon cancer progression. Clin Exp Metastasis 20: 85–90. 12650611 10.1023/a:1022502805474

[5] Oldberg A, Franzen A, Heinegard D (1986) Cloning and sequence analysis of rat bone sialoprotein (osteopontin) cDNA reveals an Arg-Gly-Asp cell-binding sequence. Proc Natl Acad Sci U S A 83: 8819–8823. 3024151 10.1073/pnas.83.23.8819 PMC387024

[6] Rodrigues LR, Teixeira JA, Schmitt FL, Paulsson M, Lindmark-Mansson H (2007) The role of osteopontin in tumor progression and metastasis in breast cancer. Cancer Epidemiol Biomarkers Prev 16: 1087–1097. 17548669 10.1158/1055-9965.EPI-06-1008

[7] Hotte SJ, Winquist EW, Stitt L, Wilson SM, Chambers AF (2002) Plasma osteopontin: associations with survival and metastasis to bone in men with hormone-refractory prostate carcinoma. Cancer 95: 506–512. 12209742 10.1002/cncr.10709

[8] Ramankulov A, Lein M, Kristiansen G, Loening SA, Jung K (2007) Plasma osteopontin in comparison with bone markers as indicator of bone metastasis and survival outcome in patients with prostate cancer. Prostate 67: 330–340. 17192877 10.1002/pros.20540

[9] Cui BK, Zhang CQ, Zhang Y, Yuan YF, Zhang YQ, et al (2006) [Osteopontin as a potential biomarker of metastasis and recurrence for hepatocellular carcinoma]. Ai Zheng 25: 876–879. 16831281

[10] Rangel J, Nosrati M, Torabian S, Shaikh L, Leong SP, et al (2008) Osteopontin as a molecular prognostic marker for melanoma. Cancer 112: 144–150. 18023025 10.1002/cncr.23147

[11] Hu Z, Xiao T, Lin DM, Guo SP, Zhang ZQ, et al (2007) [Over-expression of osteopontin in non-small cell lung cancers: its clinical significance]. Zhonghua Zhong Liu Za Zhi 29: 591–595. 18210878

[12] Hayashi C, Rittling S, Hayata T, Amagasa T, Denhardt D, et al (2007) Serum osteopontin, an enhancer of tumor metastasis to bone, promotes B16 melanoma cell migration. J Cell Biochem 101: 979–986. 17390343 10.1002/jcb.21298

[13] Zheng YH, Tian C, Meng Y, Qin YW, Du YH, et al (2012) Osteopontin stimulates autophagy via integrin/CD44 and p38 MAPK signaling pathways in vascular smooth muscle cells. J Cell Physiol 227: 127–135. 21374592 10.1002/jcp.22709

[14] Agrawal D, Chen T, Irby R, Quackenbush J, Chambers AF, et al (2003) Osteopontin identified as colon cancer tumor progression marker. C R Biol 326: 1041–1043. 14744111 10.1016/j.crvi.2003.09.007

[15] Salameh A, Dhein S (2005) Pharmacology of gap junctions. New pharmacological targets for treatment of arrhythmia, seizure and cancer? Biochim Biophys Acta 1719: 36–58. 16216217 10.1016/j.bbamem.2005.09.007

[16] Carruba G, Stefano R, Cocciadiferro L, Saladino F, Di Cristina A, et al (2002) Intercellular communication and human prostate carcinogenesis. Ann N Y Acad Sci 963: 156–168. 12095941 10.1111/j.1749-6632.2002.tb04107.x

[17] Wai PY, Mi Z, Guo H, Sarraf-Yazdi S, Gao C, et al (2005) Osteopontin silencing by small interfering RNA suppresses in vitro and in vivo CT26 murine colon adenocarcinoma metastasis. Carcinogenesis 26: 741–751. 15661802 10.1093/carcin/bgi027

[18] Moertel CG, Fleming TR, Macdonald JS, Haller DG, Laurie JA, et al (1993) An evaluation of the carcinoembryonic antigen (CEA) test for monitoring patients with resected colon cancer. JAMA 270: 943–947. 8141873

[19] Eschrich S, Yang I, Bloom G, Kwong KY, Boulware D, et al (2005) Molecular staging for survival prediction of colorectal cancer patients. J Clin Oncol 23: 3526–3535. 15908663 10.1200/JCO.2005.00.695

[20] Agrawal D, Chen T, Irby R, Quackenbush J, Chambers AF, et al (2002) Osteopontin identified as lead marker of colon cancer progression, using pooled sample expression profiling. J Natl Cancer Inst 94: 513–521. 11929952 10.1093/jnci/94.7.513

[21] Liotta LA (1992) Cancer cell invasion and metastasis. Sci Am 266: 54–59, 62–53. 10.1038/scientificamerican0292-54 1373003

[22] Trosko JE, Ruch RJ (2002) Gap junctions as targets for cancer chemoprevention and chemotherapy. Curr Drug Targets 3: 465–482. 12448698 10.2174/1389450023347371

